# A quantitative and qualitative assessment of sugar beet genotype resistance to root-knot nematode, *Meloidogyne incognita*

**DOI:** 10.3389/fpls.2022.966377

**Published:** 2023-01-11

**Authors:** Ibrahim M. A. Gohar, Amal Alyamani, Manal E. Shafi, Elshaimaa A. E. Mohamed, Rehab Y. Ghareeb, Elsayed M. Desoky, Mohamed E. Hasan, Amera F. Zaitoun, Nader R. Abdelsalam, Khaled A. El-Tarabily, Ahmed S. M. Elnahal

**Affiliations:** ^1^ Sugar Crops Research Institute, Department of Sugar Crops Disease and Pests Research, Agricultural Research Center, Giza, Egypt; ^2^ Department of Biotechnology, Faculty of Sciences, Taif University, Taif, Saudi Arabia; ^3^ Department of Biological Sciences, Zoology, King Abdulaziz University, Jeddah, Saudi Arabia; ^4^ Sugar Crops Research Institute, Department of Genetic and Breeding, Agricultural Research Center, Giza, Egypt; ^5^ Plant Protection and Biomolecular Diagnosis Department, Arid Lands Cultivation Research Institute, The City of Scientific Research and Technological Applications, New Borg El Arab, Alexandria, Egypt; ^6^ Botany Department, Faculty of Agriculture, Zagazig University, Zagazig, Egypt; ^7^ Bioinformatic Department, Genetic Engineering and Biotechnology Research Institute, University of Sadat City, Sadat City, Egypt; ^8^ Agricultural Botany Department, Faculty of Agriculture (Saba Basha), Alexandria University, Alexandria, Egypt; ^9^ Department of Biology, College of Science, United Arab Emirates University, Al Ain, United Arab Emirates; ^10^ Khalifa Center for Genetic Engineering and Biotechnology, United Arab Emirates University, Al Ain, United Arab Emirates; ^11^ Harry Butler Institute, Murdoch University, Murdoch, WA, Australia; ^12^ Plant Pathology Department, Faculty of Agriculture, Zagazig University, Zagazig, Egypt

**Keywords:** *Beta vulgaris*, *Meloidogyne incognita*, modified host-parasite index, phylogenetic analysis, quantitative and qualitative schemes, root-knot nematode, single nucleotide polymorphism, sugar beet

## Abstract

Sugar beet productivity is highly constrained by the root-knot nematode (RKN) *Meloidogyne incognita*. Eight sugar beet genotypes were screened under greenhouse conditions for their susceptibility to *M. incognita* according to an adapted quantitative scheme for assignment Canto-Saenz’s host suitability (resistance) designations (AQSCS). Besides, the degree of susceptibility or tolerance of the examined genotypes was recorded by the modified host-parasite index (MHPI) scale based on yield performance. In addition, single nucleotide polymorphism (SNP) was also determined. Sugar beet genotypes have been classified into four categories for their susceptibility or tolerance according to the AQSCS scale. The first category, the moderately resistant (MR) group implies only one variety named SVH 2015, which did not support nematode reproduction (RF≤1), and had less root damage (GI≈2). Second, the tolerant group (T) involving Lilly and Halawa KWS supported fairly high nematode reproduction (RF>1) with relatively plant damage (GI≤2). Whereas the susceptible (S) category involved four varieties, FARIDA, Lammia KWS, Polat, and Capella, which supported nematode reproduction factor (RF>1) with high plant damage (GI>2). The fourth category refers to the highly susceptible (HYS) varieties such as Natura KWS that showed (RF≤1) and very high plant damage (GI>2). However, the MHPI scale showed that Lammia KWS variety was shifted from the (S) category to the (T) category. Results revealed significant differences among genotypes regarding disease severity, yield production, and quality traits. The SVH 2015 variety exhibited the lowest disease index values concerning population density with 800/250 cm^3^ soils, RF=2, root damage/gall index (GI=1.8), gall size (GS=2.3), gall area (GA=3.7), damage index (DI=3.4), susceptibility rate (SR=2.4), and MHP index (MHPI=2.5). However, Lammia KWS showed the highest disease index values regarding population density with 8890/250 cm^3^ soils, RF= 22.2, GI= 4.8, and SR= 14.1. Meanwhile, Natura KWS the highest GS, GA and MHPI with 7.1, 8 and 20.9, respectively. The lowest DI was achieved by Capella (DI= 6) followed by Lammia KWS (DI= 5.9). For yield production, and quality traits, SVH 2015 exhibited the lowest reductions of sugar yields/beet's root with 11.1%. While Natura KWS had the highest reduction with 79.3%, as well as it showed the highest reduction in quality traits; including sucrose, T.S.S, and purity with 65, 27.3, and 51.9%, respectively. The amino acid alignment and prediction of the DNA sequences revealed the presence of five SNPs among all sugar beet verities.

## Introduction

Sugar beet (*Beta vulgaris* L.) is one of the most profitable sugar crops (Lubova et al., 2018). It is a temperate crop, providing about 35% of the world’s sugar demand since it can be sown in a varied variety of climatic conditions and is widely grown in arid regions (Wu et al., 2013; Tomaszewska et al., 2018; Bayomi et al., 2019). In Egypt, sugar beet is the second most important sugar crop cultivated in the newly reclaimed desert. Currently, sugar beet is considered the first sugar crop in Egypt cultivated in 594.248 feddans, contributing 62.2% of sugar production with an average production of 21.3 tons feddan^-1^ (GAIN, 2019).

Root knot nematodes (RKNs; *Meloidogyne* spp.) are widespread plant parasites that cause considerable damage to the cultivation of sugar beet. They play a significant role in interrupting plant physiology and limiting crop productivity (Abad et al., 2003; Al-Neami and Kahtan, 2011; Perrine-Walker, 2019; Forghani and Hajihassani, 2020). Since chemical management of RKNs using nematicides is not preferred in sugar beet production, workers have turned their focus to alternative management strategies. Interestingly, nematode management mostly relies on adequate sugar beet varieties (Reuther et al., 2017; Ashmit et al., 2021). Host resistance mainly depends on genetic and non-genetic elements (Stevanato et al., 2019; Ullah et al., 2020; Badshah et al., 2021; Al-Nemi et al., 2022).

The non-genetic element comprises cultural practices, physical processes, and chemical procedures (Davis and May, 2003; Wang et al., 2013). The genetic element includes detecting sources of resistance and using those sources in breeding programs to develop nematode-resistant genotypes (Kim and Yang, 2019; Abdelsalam et al., 2022b; Elkobrosy et al., 2022). In the agricultural ecosystem, increasing the resistance of sugar beet cultivars to nematodes can decrease nematicide application and reduce environmental pollution and production costs (Elrys et al., 2018; Kim and Yang, 2019; El-Saadony et al., 2021). Using resistant cultivars against RKNs could contribute to the effective and environmentally friendly management of crops.

In general, crop yields from resistant genotypes are higher than those from susceptible ones (Merwad et al., 2018; Ghareeb et al., 2020; Youssef et al., 2020; Ghareeb et al., 2022). Therefore, the resistant genotypes could be engaged alongside other management approaches such as organic soil improvements (Kayani et al., 2012; Abdelsalam et al., 2022a; Choudhary et al., 2022), biological control (Al-Ani, 2010; Mukhtar et al., 2013), and crop rotation using non-hosts to manage the RKNs diseases (Xie et al., 2016).

Two approaches are used to assess sugar beet varieties for susceptibility to RKNs: an adaptive quantitative scheme of Canto - Saenz’s (AQSCS) and the modified host-parasite index (MHPI). The AQSCS technique is suitable for a quick sugar beet plant host suitability test and a 60-day test, especially for the susceptible sugar beet varieties (Maareg et al., 2018). According to the AQSCS approach, host suitability is determined by using a rating system based on the gall index (GI), which serves as an indicator for plant damage, either resistant or susceptible, and the nematode reproduction factor (RF), which serves as a marker for nematode reproduction effectiveness on the host and denotes tolerance (Rady, 2021). Meanwhile, the MHPI scale could be used to standardize host suitability assessment for root-knot nematodes, which depends on the efficacy of the root’s weight and yield quality 180-days post inoculation (Maareg et al., 2018; Rady, 2021).

Molecular markers are used to investigate the structure and function of genes (Cureton et al., 2002; Kaloshian and Teixeira, 2019). Single nucleotide polymorphisms (SNPs) are molecular markers that can identify targeted gene alleles and measure substantial allelic variations (Nakitandwe et al., 2007). SNPs are naturally occurring variations among individuals that affect a single nucleotide and occur once every 1000 bases (Perkel, 2008; Dwiningsih et al., 2020). DNA markers, such as SNPs, can detect resistance genotypes, speed up the evaluation process and improve accuracy (Norouzi et al., 2015) by mapping chromosomes and tagging significant genes, as well as diversity analysis (Schneider et al., 2001; Kumar et al., 2012; Dwiningsih et al., 2020).

The present investigation evaluated various sugar beet genotypes for their disease reaction against diseases caused by RKNs and yield attributing traits to enable the development of disease-resistant and high-yield genotypes. The mechanism of resistance of these genotypes is described based on SNPs molecular markers, which differentiate between various genotypes according to SNPs among their genetic DNA sequences. We introduced resistant genotypes against RKNs with high-yielding attributes for breeders, leading to new commercial-resistant varieties. This practice is an effective eco-friendly and sustainable disease management strategy.

## Materials and methods

### Outdoor pot experiment to assess the suitability of sugar beet genotypes against RKNs, *M. incognita*

Eight sugar beet *(B. vulgaris saccharifera L.)* varieties were used in the current study, undertaken at the Sugar Crops Research Institute (SCRI), Giza, Egypt (**Table 1**). The soil was collected from Sabahia Agricultural Research Station, Alexandria, Egypt. During the first season, pots (35 cm in diameter) were filled with 5 kg sterilized soil.

Table 1Description of sugar beet *(Beta vulgaris saccharifera)* varieties assessed for their resistance to the root-knot nematode, *Meloidogyne incognita*, and their seed types (monogerm or polygerm) according to the list of varieties eligible for seed certification (OECD, 2019).

**Table:** 

Serial number	Variety	Code	Genotypes handling category	Seed type	Page
**1**	Natura KWS	A,h,DE,RS,1665	Commercial var.	monogerm	B23
**2**	FARIDA	A,ES,828	Commercial var.	polygerm	B12
**3**	Lammia KWS	A,h,DE,1665	Commercial var.	polygerm	B19
**4**	SVH 2015 #	A,m,DE,2765	Commercial var.	monogerm	B27
**5**	Lilly	A,g,FR,1881	Commercial var.	polygerm	B19
**6**	Halawa KWS	A,h,DE,1665	Commercial var.	polygerm	B15
**7**	Polat	A,h,DE,792	Commercial var.	monogerm	B25
**8**	Capella #	A,h,FR,1125	Commercial var.	polygerm	Page B8

Key symbols used in the classification of sugar beet, *Beta vulgaris* varieties are shown below;

**Table:** 

**A**	Use for sugar	**d**	Inbred	**ES**	SPAIN
**h**	2n x 2n	**e**	Diploid 2n	**FR**	FRANCE
**g**	2n + 4n	**f**	Tetraploid 4n	**#**	For deletion next year
**m**	2n x 4n	**DE**	GERMANY		
**b**	Hybrid	**RS**	SERBIA		

For the second season, plastic bags were used to sow sugar beet seeds and were filled with 10 kg of soil. This was carried out to relate plant growth and/or yield parameters for the judgment of sugar beet tested varieties for response to experienced factors after 180-200 days in the second experiment. The planting was conducted in the greenhouse at the Sabahia Agricultural Research Station, Alexandria, Egypt. Before inoculation, two weeks of healthy seedlings of sugar beet genotypes were thinned out to two plants pot^-1^.

### Identification of nematodes

Female nematodes were taken from the West Nubaria district, Egypt, and the pattern analysis was employed, using the initial individuals from single egg mass cultures of infected okra (*Abelmoschus esculentus* L.) roots with RKNs. Okra roots were analyzed and treated with a solution of triethanolamine-formaldehyde (TAF) before the examination.

Ten female nematodes were randomly selected from each okra root and the perineal patterns were cut in 45% lactic acid and added to glycerin (Hartman and Sasser, 1985). A stereomicroscope was used to analyze the perineal pattern analysis according to the procedure outlined by Singh et al. (2012).

### Nematode inoculation

To obtain a consistent source of inoculum for the current study, we undertook the mass culturing of RKNs on susceptible okra plants in the greenhouse. According to Hartman and Sasser (1985), plants were uprooted and egg masses were collected. The inoculum of nematodes was applied 1 h before injection as 4000 *M. incognita* egg pot^-1^ as recommended by Kiewnick et al. (2011), nearly 400 eggs/250 cm^3^ soils. The inoculum was divided into three punctures (around 2.5 cm deep), covered with soil, and the plants were then regularly irrigated.

### Assessment of damage index (DI)

In the first season, 60 days-post inoculation with nematode eggs, the plants were uprooted by placing the tiny pots in a sloping position into a large pan filled with water and gently shaking the pan until the holding soil was removed and the roots were rinsed. The roots were examined and ranked for galling response on a scale described by Taylor and Sasser (1978); 0 = 0 galls; 1 = 1-2 galls; 2 = 3-10 galls; 3 = 11-30 galls; 4 = 31-100 galls; 5 = 101 galls or above.

Before up-rooting the plants, 250 cm^3^ of soil around each plant was collected to a depth of 10–15 cm. Second juvenile larvae (J2) were extracted from each soil sample using a modified Bearman’s tray method as described by Barker (1985). J2 was estimated in 2 ml of each extract under the microscope ten times (20 ml) to estimate the population in 250 cm^3^ soil.

The host efficiency (RF) was determined, where RF=Pf/Pi, with Pf being the final population in 250 cm^3^ of soil and Pi being the primary inoculum. RF of less than or equivalent to one indicates no obvious increment in the nematode population (Abd-El-Khair et al., 2013). The total evaluation of the genotypes was based on Canto-Saenz’s host resistance designation scheme (Canto-Saenz, 1983), adapted by Abo-Ollo et al. (2018) as shown in **Table 2**.

**Table 2:** Adapted quantitative scheme for assignment of Canto-Saenz’s (AQSCS) host suitability (resistance) designations.

Degree of resistance (DR)	Host efficiency^z^ (RF)	Plant damage (Gall index)^y^
Resistant **(R)**	≤1	≤2
Moderately resistant **(MR)**	≤1	≈2
Tolerant **(T)**	>1	≤2
Susceptible **(S)**	>1	>2
Hyper susceptible **(HYS)**	≤1	>2

^Z^Reproduction factor: RF, ^Y^ Gall index: 0, no gall formation; 5, heavy gall formation source: Sasser et al. (1984).

### Assessment of the susceptibility to RKNs, *M. incognita* on sugar beet varieties according to the efficacy of root’s weight and a quality-based scheme of MHPI designation

**Figure 1:**
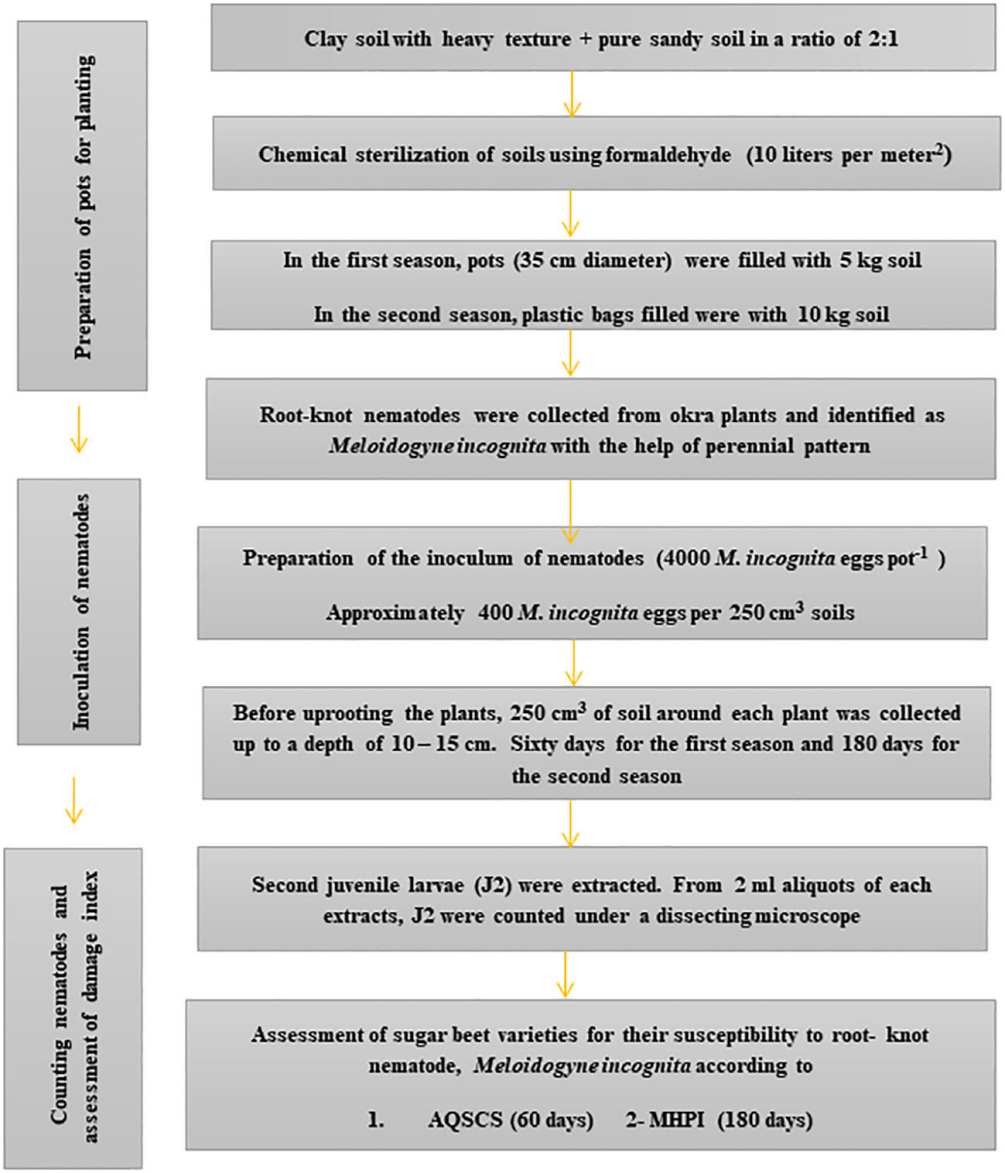
Steps of the outdoor pots experiment for sugar beet *(Beta vulgaris saccharifera)* genotypes suitability towards root-knot nematode, *Meloidogyne incognita* by two quantitative and qualitative schemes. AQSCS, adaptive quantitative scheme of Canto-Saenz’s; MHPI, modified host-parasite index.

For the second season, another set of eight pots was kept for each genotype and treated similarly to the previous season. Two pots from each variety were kept without inoculation as a control (**Figure 1**). All pots were placed in a randomized complete block design (RCBD). Six months after the inoculation of the nematode juveniles, the soil of each pot was well irrigated before removing the plant. Roots were washed in a gentle flow of tap water. Fresh weights of leaves and roots were measured. The infested plant root was investigated to determine the number of galls.

Root GI was estimated according to Sharma et al. (1994). The number of developmental stages in the root system was measured following staining with lactic acid-fuchsine. In addition, the number of *M. incognita* juveniles in pot's soils was detected and measured by sieving and using a modified Baermann-pan technique (Goodey, 1963). The technological characters were assessed in fresh roots based on total soluble solids % (TSS) using a hand refractometer, and the % sucrose was recorded following Refay (2010). The % purity was measured as a ratio between % TSS and % sucrose. Sugar root weight plant^-1^ was calculated by multiplying % sucrose by root weight.

The modified procedure was used to determine the level of resistance or susceptibility of the tested sugar beet genotypes, taking into account the impact of RKNs disease on root weight production, including roots and sugar yield. Top yield, root yield, and sugar yield plant^-1^ as additional parameters for assessing resistance to RKNs provides a comprehensive overview of the sugar beet–*Meloidogyne* interactions, resulting in a more profound process for analyzing host response according to Maareg et al. (2009). The MHPI was used to determine the susceptibility/resistance level of the tested sugar beet varieties tested for the susceptibility/resistance value, which states the amount of reduction in yield and technological characters caused by nematode infection according to the following formula:


$$\text{MHPI }=\;2\;\left[{\text{R}}_{\text{yi}}+{\text{ R}}_{\text{tech}}\right]\;÷\;\left(\text{SR }\times {\text{ P}}_{\text{yi }+\text{ tech}}\right)$$

Where:

R_yi_ = Total reduction in yield characters

R_tech_ = Total reduction in technological characters

P_yi+tech_ = Number of yield and technological characters

SR = Susceptibility rate = (RF + DI) / 2; Where: RF = reproduction factor = final population (P_f_) / initial population (P_i_) according to Oostenbrink (1966) and DI = damage index.

DI = (GI + GS + GA) / 3 according to Sharma et al. (1994). Where GS = gall size, and GA= gall area. Sugar beet varieties with MHPI ≤ 4.0 was considered tolerant (T), 4.1- 6.0 as low susceptible (LS), 6.1- 8 as moderately susceptible (MS), and ≥ 8.1 as highly susceptible (HS). Least significant differences (LSD) and a paired T- test at 0.05 and 0.01 were performed for all data.

### Molecular studies

#### DNA isolation and PCR analysis

Embryos were extracted from sugar beet grains and the DNA was isolated using Cetyl trimethyl ammonium bromide (CRAB) methods (Abd El-Fatah et al., 2020). The reaction was carried out utilizing oligonucleotide-specific primers. The reaction conditions were optimized as the following: 10µl 5x Jena Bioscience Taq Master/high yield catalogue number PCR-101S; 2 µl forward primer (25 pmol/µl^-1^); 2 µl reverse primer (25 pmol/µl^-1^); 4 µl template DNA (100 ng µl^-1^) and H_2_O µl.

Amplification was performed in a Techne Flexigene PCR thermal cycler programmed for 30 cycles as follows: 94°C/3 min (1 cycle); 94°C/30 sec, 55°C/30 sec, 72°C/1 min (30 cycles); 72°C/10 min (1 cycle); 4°C (infinitive).

#### Purification of PCR- amplified DNA

Candidate PCR bands were excised and purified from agarose gel for the subsequent steps of bioinformatics studies using an AxyPrep™ DNA Gel extraction kit (Axygen Scientific, Inc. Union City, San Francisco, California, USA), (catalogue number AP-GX-50) by the DNA gel extraction spin protocol. The agarose gel slice, including the DNA product, was then excised and transferred to a weighing boat. The gel was minced into small pieces and weighed. In this method, gel weight was considered equivalent to volume. The gel slice was transferred into 1.5 ml microcentrifuge tubes, followed by a three-fold volume of buffer DE-A (gel solubilization buffer, (Axygen Scientific, Inc.).

Vortexing was carried out to resuspend the gel in buffer DE-A, which was warmed to 75°C to completely dissolve the gel (nearly 6-8 min). Intermittent vortexing (every 2-3 min) accelerated gel solubility. The solubilized agarose was transferred into the column and centrifuged at 12000 x *g* for 1 min. The filtrate was discarded from the 2 ml microfuge tube, the AxyPrep column was restored to the 2 ml microfuge tube, and 500 µl of the wash buffer (Axygen Scientific, Inc.) was added, and centrifuged at 12000 x *g* for 30 sec. The filtrate was removed from the 2 ml microfuge tube.

The AxyPrep column was returned into to the 2 ml microfuge tube and 700 µl of buffer W2 was included, centrifuged at 12000 x *g* for 30 sec. The filtrate was discarded, and the AxyPrep column was placed into a new 2 ml microfuge tube and centrifuged at 12000 x *g* for 1 min. The AxyPrep column was then transferred into a clean tube (1.5 ml) and 30 µl of warmed H_2_O was added to the membrane center at room temperature for 1 min. For DNA elution, the tube was centrifuged at 12000 x *g* for 1 min, then the purified DNA of the excised band was checked on an agarose gel to determine the proper concentration suitable for sequencing.

#### Sequencing of PCR-amplified DNA and identification of candidate genes

The deoxyribonucleoside chain termination procedure originally developed by Grada and Weinbrecht (2013) was employed for sequencing the PCR- amplified DNA fragments. A homology search was performed using DNAMAN^®^ software (Lyon BioSoft, Quebec, Canada) and the NCBI database, National Center for Biotechnology Information, USA on the DNA level.

#### SNPs genotyping

Genomic DNA (gDNA) has been extracted from embryo tissue of all varieties utilizing the protocol reported by Saghai-Maroof et al. (1984). The quantity and quality of gDNAs were examined using a spectrophotometer and gel electrophoresis. Initially, a 600bp fragment was amplified from the genomic DNA of eight sugar beet genotypes utilizing the primers pair Nem06FWD and Nem06REV (Weiland and Yu, 2003). The concentrations were then altered to 50 ng µl^-1^ for PCR amplifications. A set of specific primer pairs (nem06FWD1, nem06REV1, NEM06FWD2, and NEM06REV2) were synthesized according to Bakooie et al. (2018) by (AnaSpec, Fremont, CA, USA) servers (**Table 3**). At the same time, other markers, Nem06, NEM06, and nem06, were projected based on accession numbers AY210437, KF303133, KF303135, and KF303134.

**Table 3:** Oligonucleotide sequences used for sugar beet resistant/susceptible genotyping to root-knot nematode *Meloidogyne incognita*.

Accession Number	Primer Name	Sequence (5’ to 3’)	Primer length	Annealing temperature (°C)	Position*^a^*	Amplicon size (bp)	Reference
AY210437.1	Nem06: F1	5’- TGCCGAGCTGCTTGACGGGTTGTC -3’	24	62.5	1-24	577	Weiland and Yu, 2003
	Nem06: R1	5’-GTTTCGCTCCTCAGAATTGCTGAAG-3’	25	59.44	577-553		
KF303133.1	nem06: F2	5’- TGACGGGTTGTCAATATGC -3’	19	55	3-21	124	Bakooie et al., 2018
	nem06: R2	5’- TCCATTTCCTGACCTACAATTAT -3’	23	57.6	126-103 ***^b^***		
KF303135.1	NEM06: F3	5’- AAAGAAAGGGAACTCAAATGTTAG -3’	24	58.3	80-103 ***^c^***	478	Bakooie et al., 2018
	NEM06: R3	5’- TCAGAATTGCTGAAGGTCATT -3’	21	55.4	557-537		

**^a^** Positions for Nem06, nem06, and NEM06 markers were predicted based on accession numbers AY210437, KF303133, and KF303135, respectively; **^b^** Allele specific primer for susceptible genotypes, **^c^** Allele specific primer for resistant genotypes.

The amplified PCR amplicons were separated on a 1% (w/v) agarose gel and bands were cut and purified from agarose gel using an AxyPrep™ DNA Gel extraction kit (Axygen Scientific, Inc.), (catalogue number AP-GX-50) and then sent for sequencing at Alfa Company, USA. The sequencing data was confirmed by NCBI BLAST search, then assembled and edited with EditSeq (DNASTAR, FinchTV). Moreover, the amplified sequence has been submitted to GenBank and has an accession number (http://www.ncbi.nlm.nih.gov).

#### Conserved regions

Multiple sequence alignments of the PCR products were performed from different genotypes utilizing the PROMALS server (Pei and Grishin, 2007), Clustal Omega server (Sievers and Higgins, 2021), and the BIOEDIT software (Hall et al., 2011), and were used to obtain the conserved regions. PROMALS was up to 30% more accurate than the best alignment algorithms, with progress for genetically different sequences.

Clustal Omega is a novel multiple sequence alignment tool that builds alignments between three or more sequences using HMM profile- algorithms and seeded guide trees. BIOEDIT 7.1.5 was a user-friendly biological sequence alignment editor that offers fundamental protein editing, alignment, analysis, and modification features.

#### Phylogenetic analysis and molecular evolution

All available nucleotide sequences related to the amplified sequence were obtained from GenBank (http://www.ncbi.nlm.nih.gov). Details on these sequences, involving their genotype, were obtained from the GenBank annotations, and a phylogenetic tree was estimated. The amplified sequences were compiled, analyzed, and aligned with those of similar sequences obtained from the NCBI GenBank database.

The neighbor-joining technique was used to infer the evolutionary history and to perform a maximum likelihood phylogenetic tree, and to determine the topology and length of tree branches. For phylogenetic analysis, Mafft server, Clustal Omega server, and MEGA7 software were used (Kumar et al., 2018; Katoh et al., 2019; Abdelsalam et al., 2022c).

### Statistical analysis

Data for both growing seasons were analyzed using the analysis of variance (ANOVA) following Mather and Jinks (1982) by MSTAT version 4 (1987). Due to the differences in the treatments, they were discriminated at a 0.05 significance level using Duncan’s Multiple Range Test (Duncan, 1955).

## Results

### Nematode identification

**Figure 2:**
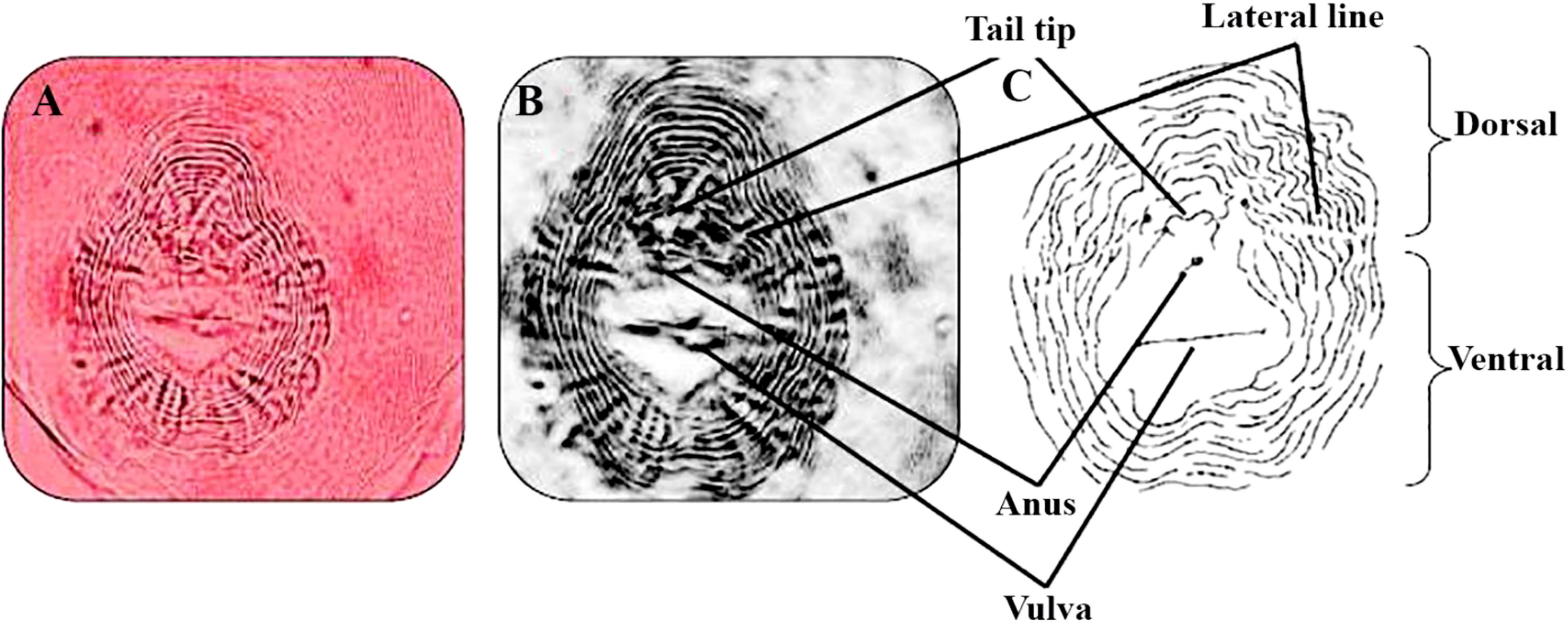
Perineal pattern of *Meloidogyne incognita*. Dissected female sections showed a characteristic oval shape of the rounded perineal pattern, typically with a high dorsal arch and usually wavy striae, which bend towards the lateral lines and may be absent from the distinct lateral field or weakly demarcated by forked striae typical of this species *M. incognita*. **(A–C)** *M. incognita* (Kofoid and White, 1919; Chitwood, 1949) perineal patterns (scale for light microscopy photos =10 μm).

The RKNs species were identified as *M. incognita* (Kofoid and White) Chitwood, with the assistance of perennial pattern according to Taylor and Netscher (1974) (**Figure 2**). A perineal pattern of a female RKNs, *M. incognita*, was photographed at the same magnification. An image processing software was used to invert images to sketch and match them with a sketch for a perineal pattern of *M. incognita* (Eisenback and Hirschmann, 1981).

### AQSCS host suitability to sugar beet - *M. incognita* interaction

For Canto-Saenz’s assignment, eight sugar beet genotypes were exposed to *M. incognita* to reproduce the host suitability test. The measurement of GI was a pointer for plant damage, and host efficiency, as well as an indication of RF. The baselines for linkage between them were used to deduce host suitability.

Four groups were detected to facilitate the assessment of sugar beet diversity in terms of RKNs host status (**Table 4**). The moderately resistant category (MR) denoted one genotype (SVH 2015), which does not sustain nematode imitation (RF≤1) and caused minor root injury (GI≈2). The next category was the tolerant group (T) involved two varieties (Lilly and Halawa KWS) that maintained comparatively high nematode reproduction (RF>1) through fairly plant damage (GI≤2 **Table 4**).

**Table 4:** Host suitability after adapted quantitative scheme of Canto-Saenz (AQSCS) for sugar beet genotypes *(Beta vulgaris saccharifera)* tested for root-knot nematode, *Meloidogyne incognita*.

Genotypes response	Sugar beet varieties	Variety Id serial number	Gall index* (GI)	J_2_/250 cm^3^ of soil	Host efficiency (RF)**
Moderately resistant (MR)	SVH 2015	4	0.8	800	2.0
Tolerant (T)	Lilly	5	3.8	400	1.0
	Halawa KWS	6	3.3	425	1.1
Susceptible (S)	FARIDA	2	1.8	2250	5.6
	Lammia KWS	3	3.8	2000	5.0
	Polat	7	3.3	1500	3.8
	Capella	8	2.8	2500	6.3
Hyper susceptible (HYS)	Natura KWS	1	1.3	1125	2.8
**Mean**			2.6	1375.0	3.4
**L.S.D. 0.05**			0.33	176.8	0.44
**SD**			1.09	817.99	2.04
**CV%**			12.86	12.86	12.86

*Gall index scale: 0 = 0 galls; 1 = 1-2 galls; 2 = 3-10 galls; 3 = 11-30 galls; 4 = 31-100 galls; 5 = 101 galls or above. **The reproduction factor (RF) was calculated as the middling final egg count distributed by 400 eggs (number of eggs with which every pot was inoculated with nearly 400 eggs/250 cm^3^ soils).

At the same time, the susceptible category (S) showed four varieties (FARIDA, Lammia KWS, Polat, and Capella), and these varieties-maintained RF>1 or RF with high plant damage (GI>2), while the highly susceptible category (HS) has only one variety (Natura KWS), which does not support nematode reproduction (RF≤1) but caused significant plant damage (GI>2 **Table 4**).

### Assessment of sugar beet variety for their susceptibility to RKNs, *M. incognita*, by MHPI

**Figure 3:**
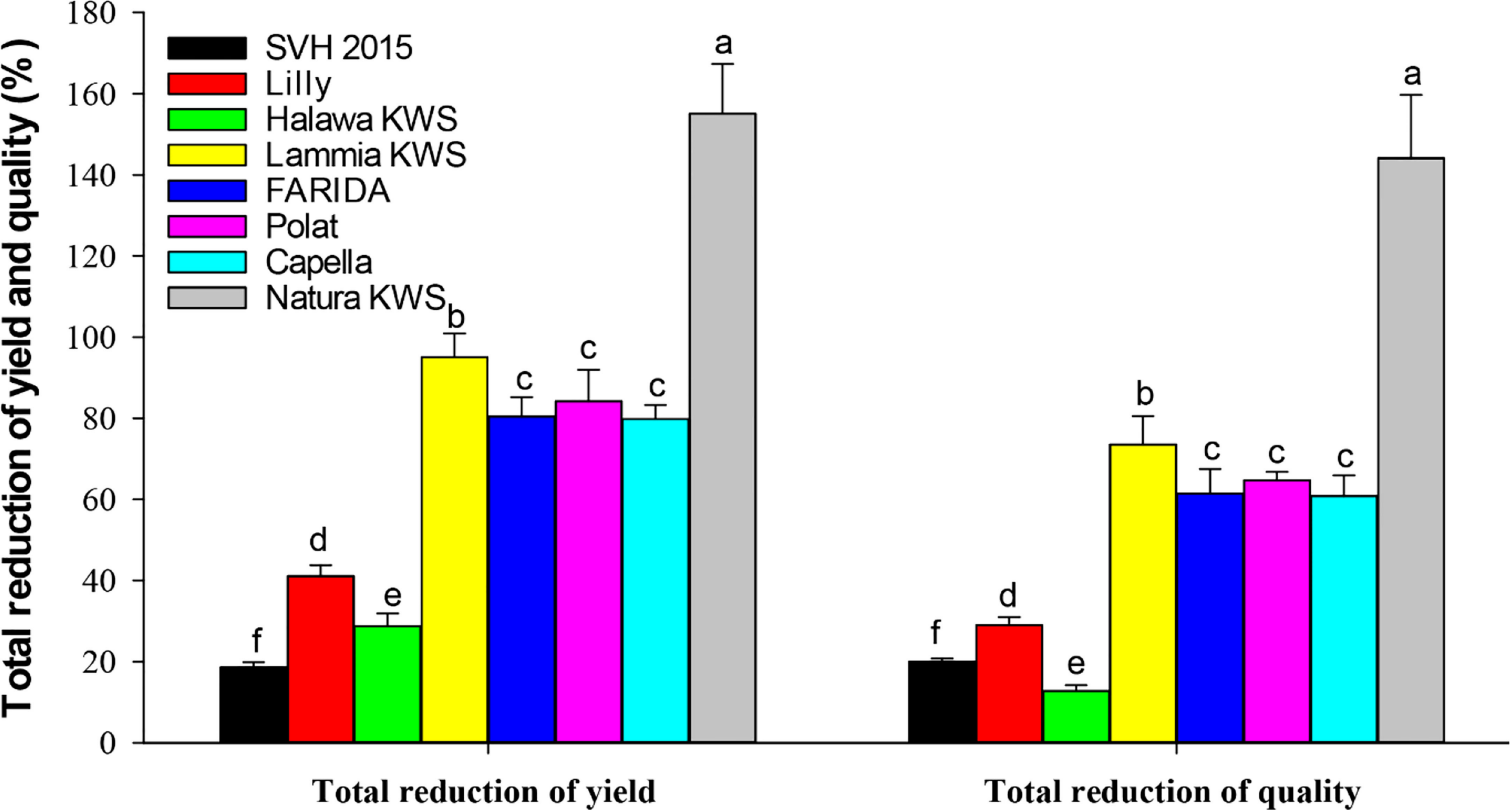
Total reduction % yield and quality characters of the screened sugar beet *(Beta vulgaris saccharifera)* varieties as influenced by root-knot nematode, *Meloidogyne incognita* infection. Mean values followed by different letters are significantly (P<0.05) different from each other according to Duncan’s Multiple Range Test.

The host suitability of the eight sugar beet varieties, *i.e.*, Natura KWS, FARIDA, Lammia KWS, SVH 2015, Lilly, Halawa KWS, Polat, and Capella to *M. incognita* infection was conducted in outdoor conditions using pots at the autumn of 2019 and terminated at spring of 2020. The results showed that *M. incognita* infection substantially influenced all quality and yield parameters of tested sugar beet genotypes (**Figure 3**; **Tables 5**, **6**).

**Table 5:** Root, top, and sugar yields of the screened sugar beet *(Beta vulgaris saccharifera)* varieties as influenced by the infection of root-knot nematode, *Meloidogyne incognita*.

Variety Id serial number	Sugar beet varieties	Root yield plant g^-1^			Top yield plant g^-1^			Sugar yield beet root^-1^			Total reduction of yield characters
		Non infested	Infested	*R %	Non infested	infested	R %	Non infested	infested	R %	
1	Natura KWS	532.4	271.5	51.0	247.4	186.0	24.8	103.3	21.4	79.3	155.1
2	FARIDA	539.5	414.9	23.1	214.5	189.6	11.6	117.6	63.8	45.7	80.4
3	Lammia KWS	620.1	447.7	27.8	253.5	218.3	13.9	117.2	54.6	53.4	95.1
4	SVH 2015	780	741.0	5.0	366.6	357.4	2.5	166.1	147.8	11.1	18.6
5	Lilly	834.6	740.3	11.3	321.8	303.5	5.7	145.2	110.3	24.1	41.1
6	Halawa KWS	776.1	715.6	7.8	312.2	300.0	3.9	166.1	137.9	17.0	28.7
7	Polat	913.9	691.8	24.3	395.2	347.0	12.2	194.7	101.7	47.7	84.2
8	Capella	1095.9	844.9	22.9	492.6	436.0	11.5	221.4	120.9	45.4	79.8
**Mean**		761.6	613.9	21.7	325.5	292.2	10.8	153.9	94.8	40.5	72.9
**L.S.D. 0.05**		96.8	78.0	2.8	41.4	37.1	1.4	19.6	12.0	5.1	9.3
**SD**		193.7	192.2	14.6	91.2	88.9	7.1	41.1	44.1	22.2	43.8
**CV%**		25.44	31.30	67.30	28.03	30.43	65.81	26.68	46.49	55.00	60.05

R %, Reduction %.

**Table 6:** Sucrose, total soluble solids (TSS), and purity percentages of the screened sugar beet varieties *(Beta vulgaris saccharifera)* as influenced by the infection of root-knot nematode, *Meloidogyne incognita*.

Variety Id serial number	Sugar beet varieties	% Sucrose			% TSS			% Purity			Total reduction of quality characters
		Nematode free	infested	R %	Nematode free	infested	R %	Nematode free	infested	R %	
**1**	Natura KWS	19.4	6.8	65.0	22	16	27.3	88.2	42.4	51.9	144.1
**2**	FARIDA	21.8	15.4	29.4	25	21	16.0	87.2	73.2	16.0	61.4
**3**	Lammia KWS	18.9	12.2	35.4	22	20	9.1	85.9	61.0	29.0	73.5
**4**	SVH 2015	21.4	19.3	9.9	24	22	8.3	89.2	87.6	1.8	20.0
**5**	Lilly	17.4	14.9	14.4	23	20	13.0	75.7	74.5	1.6	29.0
**6**	Halawa KWS	21.3	19.9	6.4	25	24.6	1.6	85.2	81.1	4.9	12.8
**7**	Polat	21.3	14.7	31.0	25	20	20.0	85.2	73.5	13.7	64.7
**8**	Capella	20.2	14.3	29.2	24	21	12.5	84.2	68.1	19.1	60.8
**Mean**		20.2	14.7	27.6	23.8	20.6	13.5	85.1	70.2	17.2	58.3
**L.S.D. 0.05**		0.05	0.04	3.5	3.0	2.6	1.7	10.8	8.9	2.2	7.4
**SD**		1.5	4.1	18.6	1.3	2.4	7.8	4.2	13.7	16.9	41.5
**CV%**		7.62	27.94	67.30	5.40	11.69	57.95	4.89	19.56	97.98	71.16

R %, Reduction %.

Significant differences (P<0.05) were found between infected and non-infected plants within screened sugar beet varieties in yield character, *i.e.*, root, top, and sugar yield plant^-1^. All the evaluated sugar beet varieties had significantly decreased in all yield characters except for the SVH 2015 variety and Halawa KWS, which had a slight reduction in sugar yield plant^-1^ (11.1% and 17.0%, respectively). Nevertheless, there was no significant decrease in root and top yields. Also, the sugar beet variety, Lilly had no significant reduction in top yield plant^-1^.

The percentage reduction in root yield plant^-1^ fluctuated from 5.0% for the SVH 2015 variety to 51.0% in Natura KWS. The top yield plant^-1^ reduction ranged from 2.5% to 24.8% for the same verities. Whereas, for sugar yield plant^-1^, the ranged reductions were more dramatic than the two other characters, varying from 11.1% for the SVH 2015 variety to 79.3% for Natura KWS, and in a total reduction of the three recorded yield characters from 18.6% in SVH 2015 variety to 155.1% in Natura KWS variety. In addition, both Lammia KWS and Polat varieties recorded 95.1% and 84.2% in the percentage of total reduction, respectively. Generally, the Natura KWS variety attained the highest reduction in top, root, and sugar yield plant^-1^ as well as the total yields, but SVH 2015 variety had the lowest ones (**Table 5**).

In addition, there were significant differences (P<0.05) found among infected and non-infected within the tested sugar beet varieties in quality characters including % sucrose, % TSS and % purity. All the tested varieties had a significant decrease in all quality characters, except for SVH 2015, Halawa KWS, and Lilly varieties, although Lilly displayed a purity of 1.6%. The reduction in the % of sucrose ranged from 9.9% in the SVH 2015 variety to 65.0% in Natura KWS, while the % of TSS ranged from 1.6% in Halawa KWS to 27.3% in Natura KWS. Reduction in % purity ranged from 1.6% to 51.9% in Lilly and Natura KWS, respectively. The total reduction of quality characters fluctuated from 12.8% to 144.1% in Halawa KWS and Natura KWS, respectively (**Table 6**).

Significant differences (P<0.05) originated in RKN final population (P_f_), GI, GS, GA, DI, and SR. The J_2_s in the soil range were 800.0- 8890.0, with SVH 2015’s variety having the lowest and FARIDA, Lammia KWS, Polat, and Capella having the highest as total nematode (Pf) values. The values of RF generally ranged, from 2.0 for SVH 2015 variety to 22.2 for Lammia KWS variety. Eventually, the varieties, FARIDA, Lammia KWS, Polat, and Capella, attained the highest RF, and SVH 2015, Natura KWS, and Halawa KWS varieties had the lowest RF values. The GI value ranged from 1.8 – 4.8 for SVH 2015, Natura KWS and Lammia KWS, and Lilly varieties, whereas the lowest values were found in SVH 2015 and Natura KWS varieties. The GS range was higher in Natura KWS and Capella with 7.1 and 6.9, respectively, whereas it was lower in SVH 2015 and Halawa KWS with 2.3 and 3.8, respectively (**Table 7**).

**Table 7:** The population density, reproduction factor, root galling symptoms, damage index, susceptibility rate, and modified host parasite index and host category of root-knot nematode *Meloidogyne incognita* on the screened sugar beet varieties *(Beta vulgaris saccharifera)*.

Variety Id serial number	Sugar beet varieties	Final population of nematodes(P_f_)/250 cm^3^ soils	Reproduction factor (RF)	Gall index(GI)	Gall size(GS)	Gall area (GA)	Damage index (DI)	Susceptibility rate (SR)	Modified host parasite index (MHP)	Host category
1	Natura KWS	1556.0	3.9	1.8	7.1	8.0	5.6	4.8	20.9	Highly susceptible (HS)
2	FARIDA	4500.0	11.3	3.2	5.8	6.3	5.1	8.2	5.8	Susceptible (S)
3	Lammia KWS	8890.0	22.2	4.8	5.9	7.0	5.9	14.1	4.0	Tolerant (T)
4	SVH 2015	800.0	2.0	1.8	2.3	3.7	3.4	2.4	2.5	Moderately resistant (MR)
5	Lilly	3022.0	7.6	4.8	5.5	5.0	5.1	6.3	3.7	Tolerant (T)
6	Halawa KWS	1200.0	3.0	2.8	3.8	3.7	3.4	2.9	3.2	Tolerant (T)
7	Polat	4833.0	12.1	3.8	5.7	7.3	5.6	8.8	5.6	Susceptible (S)
8	Capella	4333.0	10.8	3.8	6.9	7.3	6.0	8.4	5.6	Susceptible (S)
**Mean**		3972.3	9.9	3.2	5.7	6.3	5.1	7.5	6.5	
**L.S.D. 0.05**		512.5	1.3	0.4	0.7	0.8	0.7	1.0	0.8	
**SD**		2391.6	6.0	1.4	1.1	1.4	1.1	3.3	6.0	
**CV%**		60.2	60.2	43.4	18.4	22.9	21.0	44.3	92.3	

**Figure 4:**
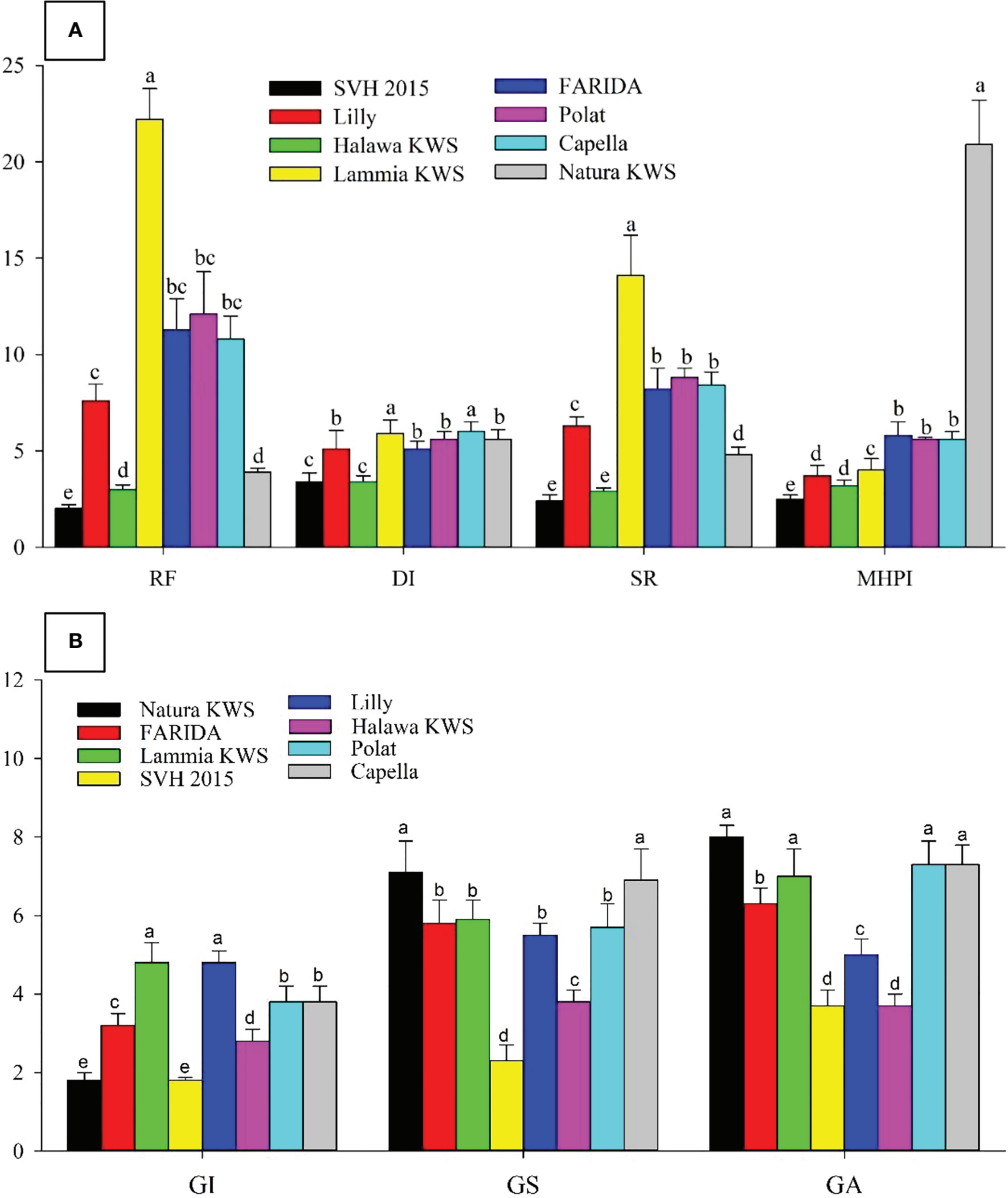
Screening sugar beet *(Beta vulgaris saccharifera)* varieties towards root- knot nematode, *Meloidogyne incognita*, using seven parameters, including **(A)**. The reproduction factor (RF), damage index (DI), susceptibility rate (SR), and modified host parasite index (MHPI); **(B)** gall index (GI), gall size (GS), and gall area (GA). Mean values followed by different letters are significantly (P<0.05) different from each other according to Duncan’s Multiple Range Test.

The GA values ranged from 3.7 in SVH 2015 to 8.0 in Natura KWS varieties. The DI range was from 3.4 for SVH 2015 and Halawa KWS varieties to 6.0 with Lammia KWS and Capella varieties. The SR values ranged from 2.4 in SVH 2015 variety to 8.8 in the Polat variety. Eventually, the variety SVH 2015 attained the lowest P_f_, RF, GI, DI, and SR values, and the Natura KWS variety had the highest at most (**Figure 4**; **Table 7**).

Analysis of resistance levels (categories) was carried out according to two screening procedures, AQSCS and MHPI. AQSCS procedure classified the eight tested sugar beet varieties into four distinguished categories; moderately resistant, tolerant, susceptible, and hyper-susceptible, almost matched with the MHPI procedure except for the number of tolerant and susceptible varieties for each (**Table 8**). Since AQSCS is a short period technique (45 -60 days), it can detect susceptible ones more precisely (Abo-Ollo et al., 2018). Still, it could not be with tolerant varieties because tolerant and resistant varieties were not dependent on the initial population (Pi) but more on yield assessment. Sugar beet tolerant varieties are expected to produce high yields regardless of nematode infection (Ashmit et al., 2021). Thus, 60 days test cannot measure yield performance. On the other hand, MHPI can detect tolerant sugar beet varieties more accurately because of its long period test (180 days) and accomplishment of yields. This explained why the sugar beet variety Lammia KWS (**Table 5**) moved from the category of susceptible ones in the AQSCS test to tolerant category varieties in the MHPI procedure (**Table 8**).

**Table 8:** Classification of different sugar beet *(Beta vulgaris saccharifera)* varieties according to susceptibility screening procedure for root-knot nematode, *Meloidogyne incognita*.

Variety Id serial number	Sugar beet varieties	Screening technique	
		Adapted Quantitative scheme of Canto- Saenz (AQSCS)	Modified host parasite index (MHPI)
1	Natura KWS	Hyper susceptible (HYS)	Hyper susceptible (HYS)
2	FARIDA	Susceptible (S)	Susceptible (S)
3	Lammia KWS	Susceptible (S)	Tolerant (T)
4	SVH 2015	Moderately resistant (MR)	Moderately resistant (MR)
5	Lilly	Tolerant (T)	Tolerant (T)
6	Halawa KWS	Tolerant (T)	Tolerant (T)
7	Polat	Susceptible (S)	Susceptible (S)
8	Capella	Susceptible (S)	Susceptible (S)

**Figure 5:**
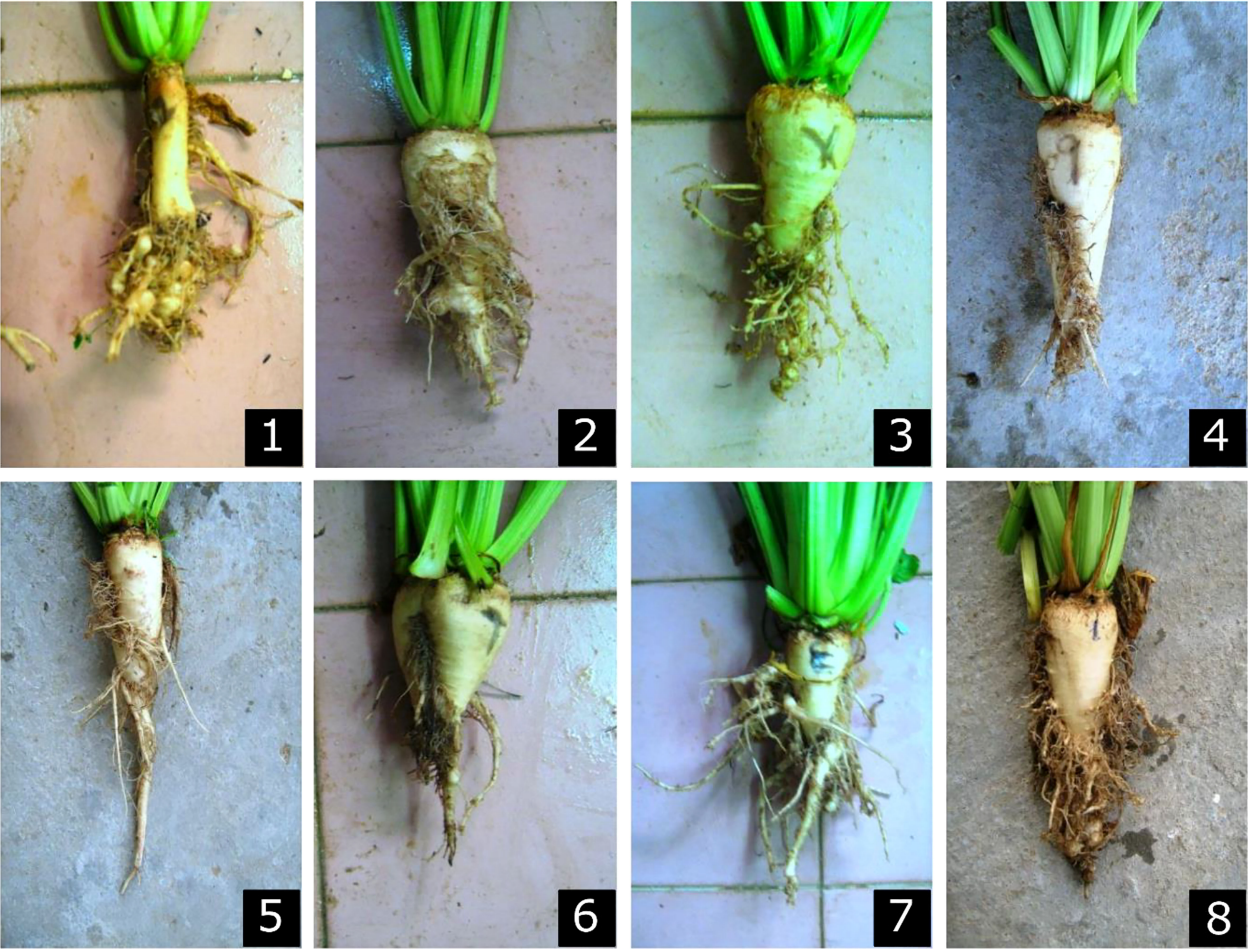
Sugar beet roots, 60-days post inoculation with *Meloidogyne incognita* in eight genotypes; (1) Natura KWS, (2) FARIDA, (3) Lammia KWS, (4) SVH 2015, (5) Lilly, (6) Halawa KWS, (7) Polat, and (8) Capella.

In addition, the comparative effect of sugar beet root symptoms 60-days post inoculation with *M. incognita* displayed differences in gall intensity and size among the eight tested genotypes (**Figure 5**).

### Analysis of SNPs markers

**Figure 6:**
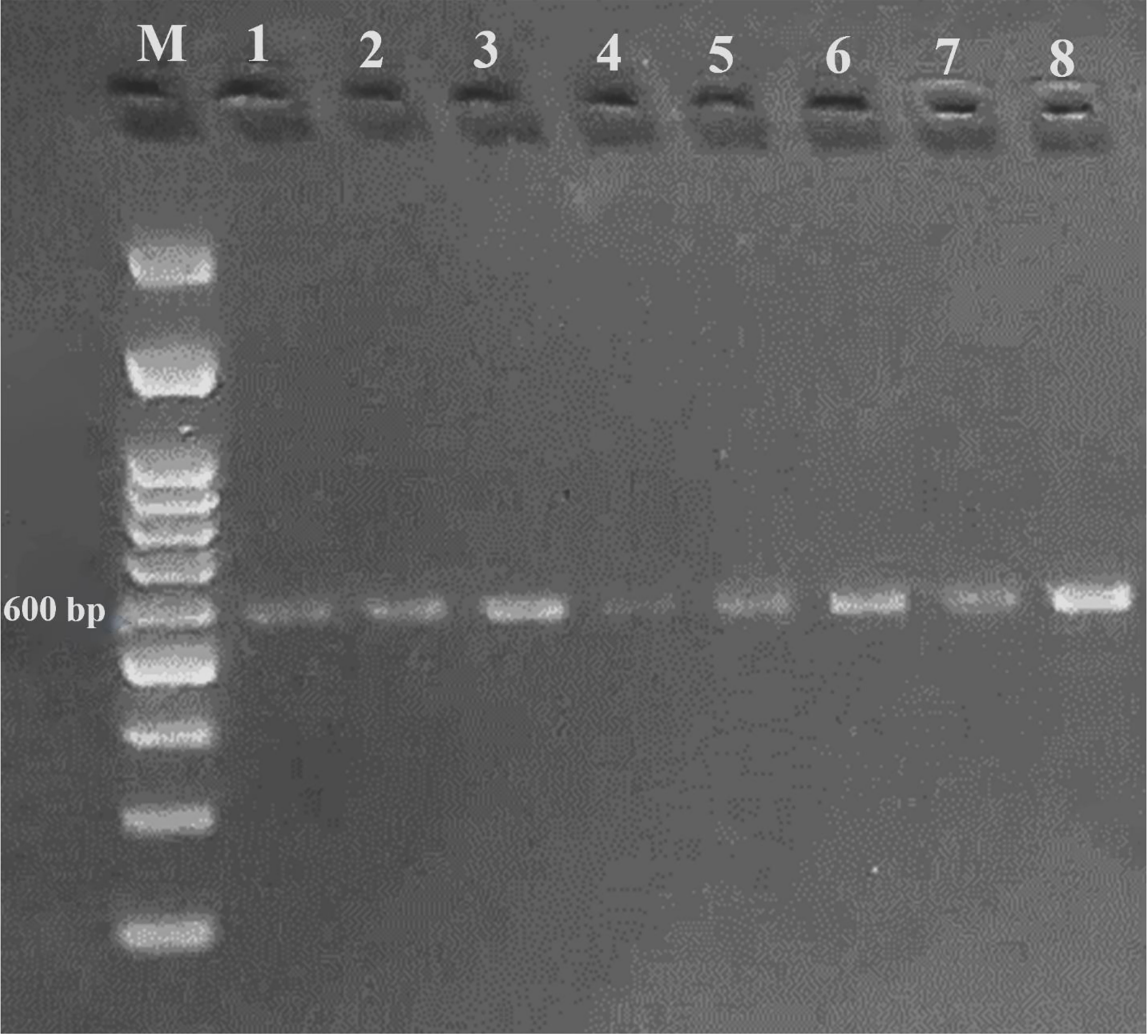
Agarose gel electrophoresis of PCR product amplified from eight sugar beet genotypes genomic DNA using Nem06FWD and Nem06REV specific primer.

**Figure 7:**
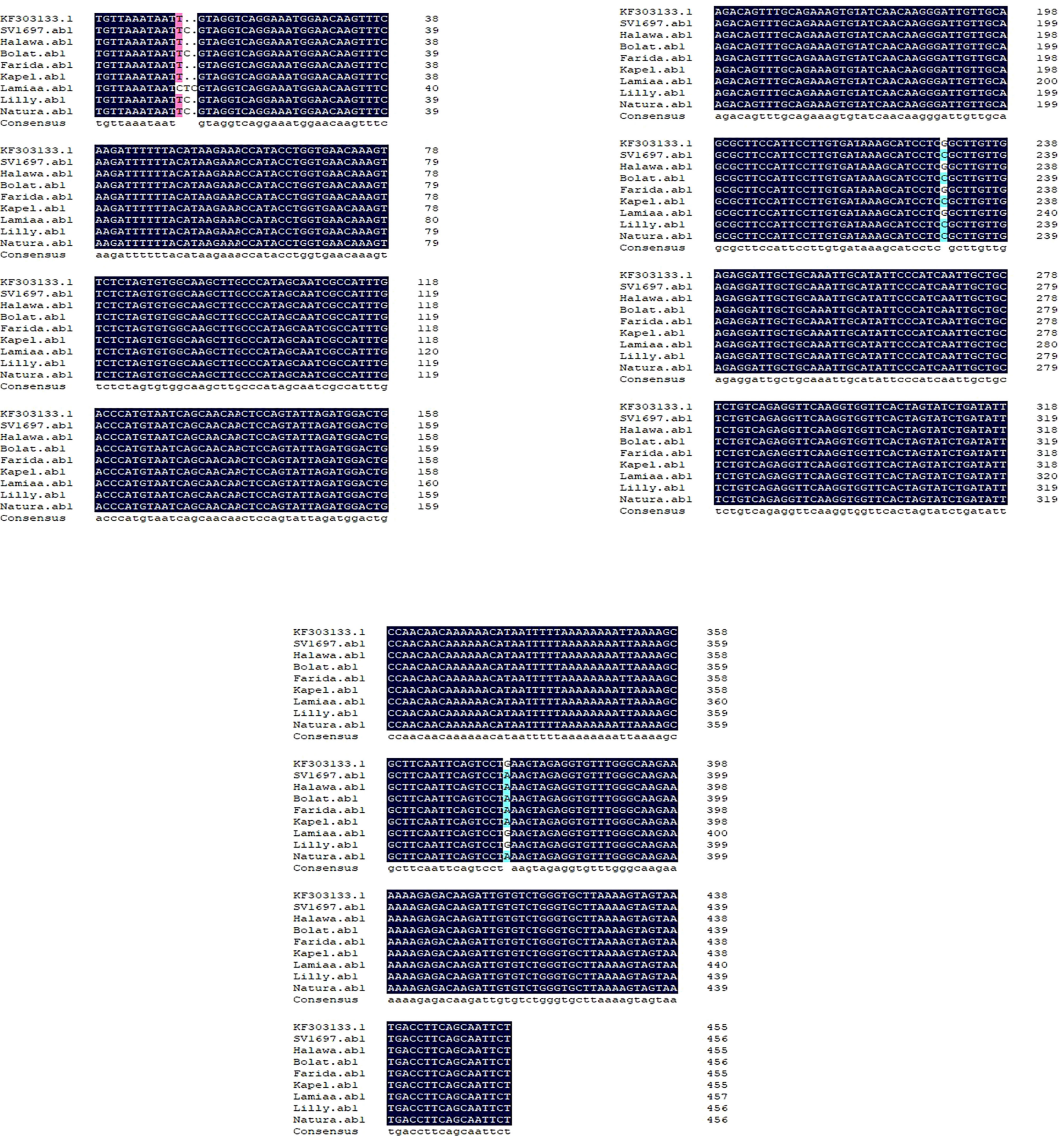
Single nucleotide polymorphisms (SNPs) detection for Anchor KF303133.1 and eight sugar beet genotypes sequences by using the DNAMAN**^®^** software (Lyon BioSoft, Quebec, Canada). Gaps in the sequences are indicated by dots ‘‘.’’, showing the conserved consensus sequences at the last sequence. SNPs are indicated by different shading colors.

**Figure 8:**
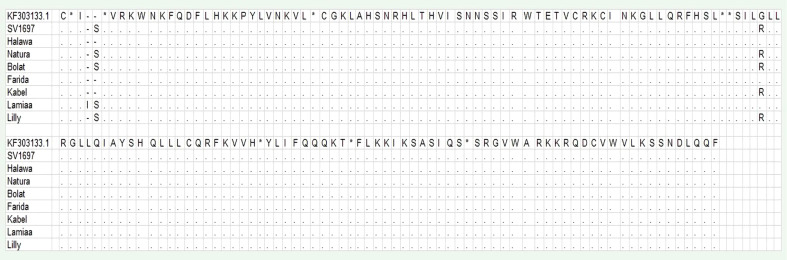
Alignment of the deduced amino acid sequences of eight obtained with MEGA-X software. Conserved consensus sequences are indicated by dots.

A DNA fragment of 600 bp was amplified in eight sugar beet varieties using a combination of Nem06REV and Nem06FWD primers (**Figure 6**). The homology search on the GenBank database of this fragment with sequence (KF303133.1) showed 99.47% similarity among the sequences. Alignment and amino acid composition prediction of the DNA sequences by MEGA-X and DNAMAN software revealed the presence of five SNPs among them (**Figures 7**, **8**).

The genotype Lamiaa sequence had a transversion in site number 12 (T→C), leading to the insertion of isoleucine. At site number 13, there was an insertion of C in SV1697, Halawa, Natura, Bolat, and Lilly and an insertion of T in genotype Lamiaa. This insertion led to the insertion of serine in SV1697, Natura, Bolat, Lamiaa, and Lilly. An insertion of C was detected in site number 14 of the Lamiaa sequence. At site number 232, a transversion (G→C) in SV1697, Bolat, Kabel, Natura, and Lilly sequences replaced glycine with arginine. A guanine base was transitioned with adenine in site number 377 in Lamiaa and Lilly sequences with no predicted amino acid changing in response to this transition.

### Molecular evolution and phylogenetic analysis

**Figure 9:**
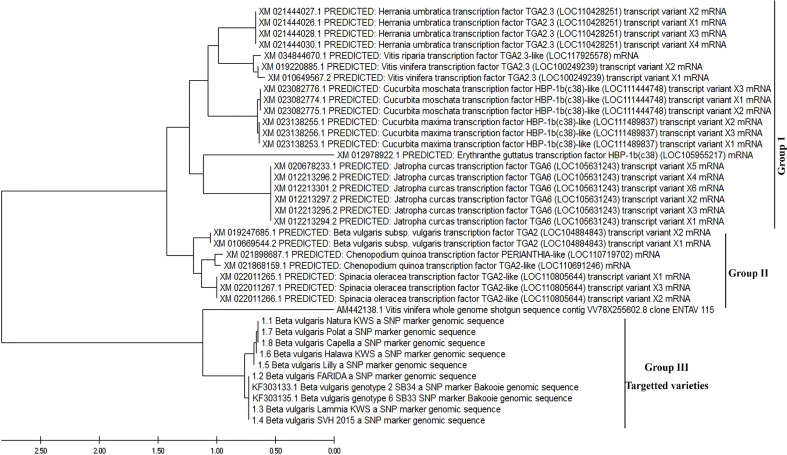
Molecular evolutionary and phylogenetic analysis was inferred using the Maximum Likelihood method and Tamura-Nei model.

We established a PCR-based method to evaluate the SNPs for resistance to RKNs among sugar beet genotypes. PCR with confronting 2-pair primers (PCR-CTPP) method simultaneously monitored polymorphism at two independent SNPs in one tube. The complete open reading frame of the eight queries cloned gene sequences from eight varieties and two genotypes retrieved from GenBank (**Figure 9**) were used to carry out a phylogenic analysis. Evolutionary distances were calculated and subjected to construct the maximum-likelihood tree. It was found that the tree was divided into two groups: the predicted *B. vulgaris* subsp. *vulgaris* transcription factor which was divided apparently from the eight genotypes into two genotypes, 2 and 6. The maximum-likelihood tree revealed that the eight varieties of *B. vulgaris* were grouped and were closely related to the two genotypes, *B. vulgaris* genotype 2_SB34 and *B. vulgaris* genotype 6_SB33. On the other hand, these varieties were distantly related to all the predicted sequences of *B. vulgaris* subsp. *vulgaris* transcription factor **(** **Figure 9**).

For ease, the sum of “r” values equals 100. Rates of unlike transitional substitutions are displayed in bold and those of transversion substitutions are displayed in italics (**Table 9**). The nucleotide frequencies were 29.38% (A), 28.09% (T/U), 19.89% (C), and 22.65% (G). This assessment involved 38 nucleotide sequences. For each sequence pair, all unclear locations were removed (pairwise deletion option). The previous dataset has a set of 43692 positions. MEGA X was used to conduct evolutionary analyses.

**Table 9:** Maximum composite likelihood estimate of the pattern of nucleotide substitution.

	A	T	C	G
**A**	–	*1.58*	*1.12*	**25.68**
**T**	*1.66*	–	**12.33**	*1.28*
**C**	*1.66*	**17.41**	–	*1.28*
**G**	**33.31**	*1.58*	*1.12*	–

Each entry shows the probability of ***substitution*** (r) from one base (row) to another base (column). Rates of unlike transitional substitutions were displayed in bold and those of transversion substitutions are displayed in italics.

The substitution pattern and the rate were assessed following the Tamura and Nei (1993) model (**Table 10**). The number of diverse transitional substitutions is displayed in bold, while those of transversions are presented in italics. The relative values of instantaneous “*r”* should be considered when assessing them. For simplicity, the sum of “*r”* values was made equal to 100, The nucleotide frequencies are “A” = 30.64%, T/U = 29.63%, C = 19.00%, and G = 20.73%. Tree topology was automatically generated to estimate ML values (**Figure 9**). The highest Log-likelihood for this computation was -78009.775. This examination included 38 nucleotide sequences.

**Table 10:** Maximum likelihood estimate of substitution matrix of nucleotide.

	A	T/U	C	G
**A**	–	*7.61*	*4.88*	**9.23**
**T/U**	*7.87*	–	**10.06**	*5.33*
**C**	*7.87*	**15.69**	–	*5.33*
**G**	**13.64**	*7.61*	*4.88*	–

*Each entry is the probability of ***substitution*** (r) from one base (row) to another base (column). Rates of unlike transitional substitutions were displayed in bold and those of transversion substitutions are displayed in italics.

The substitution pattern and the rate were estimated following the Jones et al. (1992) model (**Table 11**). The relative value of instantaneous “*r”* should be considered. The sum of “ *r”* values was rated equal to 100. The amino acid frequencies were 7.69% (A.), 5.11% (R.), 4.25% (N.), 05.13% (D.), 02.03% (C.), 4.11% (Q.), 6.18% (E.), 7.47% (G.), 2.30% (H.), 05.26% (I.), 09.11% (L.), 05.95% (K.), 02.34% (M.), 04.05% (F.), 5.05% (P.), 6.82% (S.), 05.85% (T.), 1.43% (W.), 03.23% (Y.), and 6.64% (V.). Tree topology was automatically generated to estimate ML values. This calculation has a maximum log-likelihood of -49914.525. This investigation included 38 amino acid sequences. The final dataset had a total of 13569 positions.

**Table 11:** Maximum likelihood estimate of substitution matrix of deduced amino acid sequences.

	A	R	N	D	C	Q	E	G	H	I	L	K	M	F	P	S	T	W	Y	V
**A**	–	0.14	0.12	0.22	0.06	0.12	0.34	0.67	0.03	0.10	0.15	0.11	0.06	0.03	0.51	1.37	1.39	0.01	0.02	1.01
**R**	0.21	–	0.10	0.04	0.11	0.64	0.10	0.53	0.38	0.07	0.18	2.01	0.05	0.01	0.19	0.35	0.20	0.09	0.04	0.06
**N**	0.22	0.12	–	1.48	0.03	0.16	0.19	0.30	0.48	0.13	0.06	0.78	0.04	0.02	0.03	1.79	0.71	0.00	0.12	0.06
**D**	0.33	0.04	1.22	–	0.01	0.11	2.49	0.49	0.12	0.03	0.03	0.09	0.02	0.01	0.03	0.21	0.13	0.00	0.08	0.11
**C**	0.23	0.27	0.07	0.03	–	0.02	0.02	0.21	0.09	0.04	0.08	0.02	0.05	0.14	0.03	0.76	0.14	0.08	0.35	0.21
**Q**	0.22	0.80	0.17	0.14	0.01	–	1.09	0.09	0.68	0.02	0.33	0.91	0.06	0.01	0.42	0.19	0.16	0.01	0.04	0.06
**E**	0.43	0.08	0.13	2.07	0.01	0.73	–	0.43	0.03	0.03	0.05	0.53	0.02	0.01	0.05	0.11	0.10	0.01	0.01	0.16
**G**	0.69	0.36	0.17	0.34	0.06	0.05	0.36	–	0.02	0.01	0.03	0.08	0.02	0.01	0.05	0.66	0.10	0.04	0.01	0.16
**H**	0.09	0.85	0.89	0.27	0.08	1.21	0.08	0.08	–	0.05	0.26	0.16	0.04	0.10	0.30	0.26	0.14	0.01	0.98	0.04
**I**	0.14	0.06	0.11	0.03	0.02	0.02	0.04	0.02	0.02	–	1.10	0.06	0.59	0.16	0.03	0.14	0.77	0.01	0.05	3.28
**L**	0.12	0.10	0.03	0.02	0.02	0.15	0.03	0.03	0.06	0.64	–	0.05	0.47	0.53	0.28	0.21	0.08	0.04	0.04	0.61
**K**	0.15	1.73	0.56	0.08	0.01	0.63	0.55	0.10	0.06	0.06	0.07	–	0.08	0.01	0.06	0.17	0.29	0.01	0.01	0.04
**M**	0.19	0.11	0.07	0.05	0.04	0.10	0.06	0.05	0.04	1.32	1.82	0.19	–	0.09	0.04	0.10	0.64	0.01	0.03	1.05
**F**	0.06	0.02	0.02	0.01	0.07	0.01	0.01	0.02	0.05	0.21	1.18	0.01	0.05	–	0.04	0.33	0.04	0.04	0.92	0.20
**P**	0.78	0.19	0.03	0.03	0.01	0.34	0.06	0.08	0.14	0.03	0.50	0.07	0.02	0.03	–	0.99	0.36	0.01	0.02	0.07
**S**	1.55	0.27	1.12	0.16	0.23	0.12	0.10	0.73	0.09	0.11	0.28	0.15	0.03	0.20	0.73	–	1.45	0.02	0.11	0.14
**T**	1.83	0.17	0.52	0.11	0.05	0.11	0.11	0.12	0.06	0.70	0.13	0.30	0.26	0.03	0.31	1.69	–	0.01	0.03	0.39
W	0.03	0.33	0.01	0.02	0.12	0.04	0.04	0.21	0.02	0.04	0.25	0.03	0.02	0.11	0.02	0.11	0.02	–	0.13	0.08

Models with the lowest bayesian information criterion (BIC) score are thought to define the substitution pattern better. **Table 11** additionally includes maximum likelihood values (lnL), “AICc” values (Akaike Information Criterion, corrected), and the number of parameters (including branch “lengths”) for each model. Non-uniformity of the evolutionary rate between sites might be modeled using a discrete gamma distribution (+*G*) with five categories and the assumption that a specific fraction of sites was evolutionarily invariant “+*I*”.

**Table 12** displayed proper estimations of gamma shape parameters and/or the assumed fraction of the invariable site. The estimated or assumed values of transition or transversion bias “R” are also presented for each model. Each nucleotide pair was followed by nucleotide frequency “*f*” and base substitution rates “*r*”. When assessing them, relative values of instantaneous *r* would be considered. The sum of r values for each model was set to (1). A tree topology was automatically constructed to estimate “ML” values. Ten nucleotide sequences were examined in this study. The final dataset had 915 positions.

**Table 12:** Maximum likelihood fits of 24 different nucleotide substitution models.

Model	Parameters	BIC	AICc	*InL*	(*+l*)	(+G)	R	f(A)	f(T)	f(C)	f(G)	r(AT)	r(AC)	r(AG)	r(TA)	r(TC)	r(TG)	r(CA)	r(CT)	r(CG)	r(GA)	r(GT)	r(GC)
HKY	21	14858.071	14716.234	-7337.044	n/a	n/a	0.52	0.330	0.268	0.178	0.225	0.087	0.58	0.078	0.108	0.062	0.073	0.108	0.093	0.073	0.114	0.087	0.058
HKY+I	22	14867.153	14718.568	-7337.205	0.00	n/a	0.51	0.330	0.268	0.178	0.225	0.088	0.059	0.076	0.109	0.060	0.074	0.109	0.091	0.074	0.112	0.092	0.059
TN93	22	14867.842	14719.257	-7337.549	n/a	n/a	0.44	0.330	0.268	0.178	0.225	0.092	0.061	0.057	0.114	0.060	0.077	0.114	0.102	0.077	0.084	0.086	0.061
HKY+G	22	14868.631	14720.046	-7337.944	n/a	8.58	0.055	0.330	0.268	0.178	0.225	0.086	0.057	0.081	0.106	0.064	0.072	0.106	0.096	0.072	0.118	0.086	0.057
TN93•1	22	14874.226	14718.895	-7336.361	0.00	n/a	0.53	0.330	0.268	0.178	0.225	0.086	0.057	0.066	0.106	0.078	0.072	0.106	0.117	0.072	0.096	0.086	0.057
TN93+G	23	14876.472	14721.141	-7337 484	n/a	27.40	0.54	0.330	0.268	0.178	0.225	0.086	0.060	0.067	0.106	0.00	0.070	0.106	0.117	0.072	0.098	0.086	0.057
HKY•G•I	23	14877.677	14722.346	-7338.086	0.00	8.67	0.48	0.330	0.268	0.178	0.225	0.090	0.060	0.073	0.111	0.58	0.076	0.111	0.87	0.076	0.108	0.090	0.060
GTR	25	14881 .143	14712.320	-7331.058	n/a	n/a	0.33	0.330	0.268	0.178	0.225	0.139	0.095	0.050	0.172	0.055	0.041	0.176	0.083	0.038	0.073	0.049	0.030
TN93•G•I	24	14883.558	14721.481	-733.646	0.00	200.00	0.41	0.330	0.268	0.178	0.225	0.093	0.062	0.051	0.115	0.070	0.079	0.115	0.106	0.079	0.075	0.093	0.062
GTR+G	26	14891.780	14716.213	7331.996	n/a	200.00	0.49	0.330	0.268	0.178	0.225	0.131	0.108	0.082	0.162	0.055	0.027	0.200	0.083	0.000	0.0121	0.32	0.000
GTR•	26	14894.101	14718.534	7333.156	0.00	n/a	0.050	0.330	0.268	0.178	0.225	0.137	0.088	0.0860	0 169	0.054	0.032	0.164	0.081	0.015	0 126	0.038	0.012
GTR +G+I	27	14903.938	14721.627	-7333.694	0.00	200.00	0.36	0.330	0 268	0.178	0.225	0.156	0.060	0.066	0 192	0.043	0.029	0.110	0.064	0.083	0.097	0.034	0.066
T92	19	14906.958	14778.617	-7370.249	n/a	n/a	0.62	0.299	0.299	0.201	0.201	0.091	0.061	0.079	0.091	0.079	0.061	0.091	0.117	0.061	0.117	0.091	0.061
T92+1	20	14915.829	147780.740	-7370.304	0.00	n/a	0.61	0.299	0.299	0.201	0 201	0.091	0.061	0.078	0.091	0.078	0.061	0.091	0 116	0.061	0.116	0.091	0.061
T92•G	20	14916.344	14781.254	-7370.561	n/a	51.98	0 61	0.299	0.299	0.201	0.201	0.091	0.062	0.078	0.091	0.078	0.062	0.091	0 116	0.062	0.116	0.091	0.062
T92+G+I	21	14925.151	14956.314	-7370.584	0.00	100.19	0.060	0.299	0.299	0.201	0.225	0.091	0.062	0.077	0.092	0.077	0.062	0.092	0 114	0.062	0.114	0.092	0.062
JC	17	15070.096	14955.254	-7460.579	n/a	n/a	0.50	0.250	0.250	0.250	0.250	0.083	0.083	0.083	0.083	0.083	0.083	0.083	0.083	0.083	0.083	0.083	0.083
K2	18	15078.284	14956.692	-7460.292	n/a	n/a	0.42	0.250	0.250	0.250	0.250	0.088	0.088	0.074	0.088	0.074	0.088	0.088	0.074	0.088	0.074	0.088	0.083
JC•I	18	15078.867	14957.275	-7460584	0.00	n1a	0.050	0.250	0.250	0.250	0.250	0.083	0.083	0.039	0.083	0.083	0.083	0.083	0.083	0.083	0.083	0.083	0.083
JC•G	18	15079.007	14957.415	-7460.654	n/a	200.00	0.050	0.250	0.250	0.250	0.250	0.083	0.083	0.8383	0.083	0.083	0.083	0.083	0.083	0.083	0.083	0.083	0.083
K2•1	19	15087.044	14958.703	-7460.292	0.00	n/a	0.42	0.250	0.250	0.250	0.250	0.08	0.088	0.074	0.086	0.074	0.088	0.088	0.074	0.088	0.074	0.088	0.088
K2•G	19	15087.186	14958.845	-7460.363	n/a	200.00	0.42	0.250	0.250	0.250	0.250	0.086	0.088	0.074	0.088	0.074	0.088	0.088	o 074	0.088	0.074	0.088	0.088
JC+G•I	19	15087.769	14959.428	-7460.654	0.00	200.00	0.050	0.250	0.250	0.250	0.250	0.083	0.083	0.083	0.083	0.083	0.083	0.083	0.083	0.083	0.083	0.083	0.083
K2+G+I	20	15095.960	14960.871	-7460.369	0.00	200.00	0.42	0.250	0.250	0.250	0.250	0.088	0.088	0.074	0.088	0.074	0.088	0.088	0.074	0.088	0.074	0.088	0.088

Each entry is the probability of substitution (r) from one amino acid (row) to another (column).

### Estimation of the maximum likelihood of transition or transversion bias

The calculated transition/transversion bias “*R”* was 0.45. Kimura’s two-parameter model assessed substitution patterns and rates (1980). The nucleotide frequency was rated as A = 25.00%, C = 25.00%, T/U = 25.00%, and G = 25.00%. A tree topology was computed automatically to estimate the value of “ML”. For this computation, the highest log-likelihood was -7460.435. This study included ten nucleotide sequences. The final dataset had a total of 915 positions (**Table 13**).

**Table 13:** Composite distance of the query sequence of eight varieties with genotype on databases.

	1.1	1.2	1.3	1.4	1.5	1.6	1.7	1.8	KF303133.1	KF303135.1
1.*_Beta_vulgaris_*Natura_KWS_a_SNP_marker										
1.2*_Beta_vulgaris_*FARIDA_a_SNP_marker	1.85342									
1.3*_Beta_vulgaris_*Lammia_KWS_a_SNP_marker	0.04114	2.31767								
1.4*_Beta_vulgaris_*SVH_2015_a_SNP_marker	0.02309	1.58318	0.00180							
1.5*_Beta_vulgaris_*Lilly_a_SNP_marker	0.91332	0.82090	1.01105	1.08775						
1.6*_Beta_vulgaris_*Halawa_KWS_a_SNP_marker	1.03339	0.53543	1.14599	0.78442	0.04382					
1.7*_Beta_vulgaris_*Polat_a_SNP_marker	0.01610	1.97368	0.04433	0.05586	0.92634	1.29015				
1.8*_Beta_vulgaris_*Capella_a_SNP_marker	0.04082	2.43249	0.04972	0.06929	1.10536	1.67552	0.02394			
KF303133.1*_Beta_vulgaris_*genotype_2_SB34_a_SNP_marker_Bakooie	0.06680	2.30083	0.04864	0.04950	1.20690	2.05020	0.01362	0.00584		
KF303135.1*_Beta_vulgaris_*genotype_6_SB33_SNP_marker_Bakooie	0.04912	2.22614	0.03502	0.03564	1.11359	1.94378	0.00973	0.00195	0.00194	

## Discussion

According to the results of the present study, eight sugar beet genotypes were assigned for host suitability towards RKNs *M. incognita* using two quantitative approaches, including AQSCS and MHPI, and utilizing the molecular marker SNPs to differentiate between genotypes. This investigation aimed to categorize sugar beet varieties as tolerant, susceptible, or resistant. KRNs reproduction on tolerant sugar beet varieties was low, and the most tested tolerant varieties were susceptible but gave high yields.

Within each category there were significant differences at (P<0.05) in the AQSCS technique of host suitability of sugar beet genotypes against *M. incognita*. The GI and RF were the main tools used to analyze host suitability for sugar beet varieties. The obtained results were in harmony with those reported by Abd-El-Khair et al. (2013) and Ansari et al. (2019), displaying various levels of sugar beet susceptibility to *M. incognita* based on their DI.

According to Abo-Ollo et al. (2018), sugar beet varieties were classified into three groups based on the host suitability test. The first group included susceptible genotypes with root GI>2 and RF>2. The second category in this concern was sugar beet varieties with root GI≤2 and RF>2 and the third classification hold only one genotype with the lowest RF and the highest root GI *i.e.*, the hyper-susceptible one, which was dependent on the combination of GI and RF. Dividing the final population (Pf) by the initial population (Pi) resulted in the (RF) (Oostenbrink, 1966), which expressed the capability of a crop variety to multiply or diminish a certain Pf.

Values more than “1” indicated nematode reproduction by a crop variety, and the variety was designated as “susceptible”. Meanwhile, a value less than “1” indicated nematode decline through crop varieties; and it was designated as “resistant”. This concept of resistance differs considerably from the definition of resistance e.g., microbial pathogens, which was grounded on the potential response of a host and the consequence on the pathogen (Bose et al., 2018; Akhtar et al., 2022).

Reuther et al. (2017) stated that according to the official German seed registration, successful candidate varieties for registration must have enhanced traits and “yield” which is the key trait that must be enhanced. However, supplementary traits such as exact resistance to a pathogen can also be measured as an improvement. In this regard, the currently existing varieties with resistance to RKNs have been registered. These trials were currently achieved in pots under greenhouse conditions. Two weeks after the termination of the trial, the Pf/Pi value was investigated from a sub-sample of 800 g of soil (out of three pots representing one replicate). A variety was approved as resistant when the Pf/Pi value was <1 in four replications.

Abo-Ollo et al. (2018) evaluated seventeen sugar beet genotypes for resistance against RKNs utilizing the DI that relies on root GI and RF. Sugar beet genotypes were categorized as follows; 4 were resistant (R), 3 were moderate resistant (MR), 6 were tolerant (T), 2 were susceptible (S), and 2 were hyper susceptible (HYS). The results from the current study were consistent with those of Maareg et al. (2009) who studied nematode reproduction on tolerant varieties and found that all tolerant varieties displayed less potential to support RKNs reproduction than the susceptible variety.

Despite the importance of the scales in expressing the differences in the degrees of nematode development, RF, and DI, the results indicated that these scales do not take into consideration the evaluation of real damage occurring in plant growth, yield, and quality characters of nematode infected sugar beet plants. It can nevertheless still be utilized in the fast-screening procedure as the maximum time it takes was 60 days. On the other hand, the MHPI scale has also been used to quickly standardize sugar beet host suitability resistance to RKNs. In the scorned protocol used in the current study at P<0.05, statistical differences were found among the different sugar beet varieties that greatly varied in their susceptibility/resistance to infection with *M. incognita*. Thus, they could be classified according to the MHPI scale (Maareg et al., 2018) into four significantly distinguished groups as follows; 1 (MR) genotype including SVH 2015, 3 (T) genotypes including Lammia KWS, Lilly and Halawa KWS, 3 (S) genotypes including FARIDA, Polat, and Capella, and 1 (HYS) including Natura KWS.

The results from the current study also showed that *M. incognita* infection significantly affected the quality and yield characteristics of the evaluated sugarbeet genotypes. Also, the genus *Meloidogyne* has many species that negatively affected yield such as *M. graminicola* which may reduce rice yields by up to 80% and its life cycle can be completed within 19 days under ideal environmental conditions (Bernard et al., 2017; Mantelin et al., 2017). Furthermore, the infected genotypes showed considerable differences in symptoms regarding root DI, GI, RF, final population, and SR susceptibility rate, which were consistent with those observed by Maareg et al. (2009). Despite the importance of the scales in expressing the differences in the degrees of nematode development, Rf, and DI, these scales do not take into consideration the evaluation of real damage occurring in plant growth, yield, and quality characteristics of infected sugar beet (Abd-El-Khair et al., 2013; Abo-Ollo et al., 2018).

Otherwise, El-Nagdi and Youssef (2016) evaluated certain sugar beet genotypes for their resistance/susceptibility against *M. incognita*, rendering host vigor estimated as an average of root and leaf weight potentials (total yield potential) and quality characters. Results showed a variation in resistance depending on this scale. Since Bayomi et al. (2019) assessed some sugar beet based on the MHPI scale, which expresses the amount of damage rather than nematode symptoms in both plant growth (in dry weight of leaves and roots and diameter of the root) and quality characters. However, these scales do not consider the real damage in roots, top and sugar yields, and production characters in tested varieties. On the other hand, the MHPI scale used by Maareg et al. (2009 and 2018) was more suitable for evaluating the sugar beet-*Meloidogyne* interactions based on the degree of susceptibility/resistance. The MHPI was calculated by dividing the percentage of gross average reduction in all quality and yield parameters by the susceptibility rate. It could be ranked as a standardization of host suitability technique for sugar beet resistance against RKNs.

Based on the MHPI technique, the screened sugar beet genotypes against RKNs were classified into three groups as follows; a) four (T) genotypes including Lammia KWS, SVH 2015, Lilly and Halawa KWS; b) three (S) genotypes including Capella, Polat, and FARIDA; c) one (HYS) genotype including Natura KWS. This scale was more suitable because of the generally high correlation between the percentage reduction in total yield, quality characters, and crop damage. Using the reduction in roots and sugar yields as well as sucrose, % TSS and % purity was also very important as they affected the suitability of sugar beet varieties for farmers and sugar companies.

Due to the absence of effective eco-friendly nematode management methods, developing sugar beet resistant to RKNs is highly desirable (Yu, 1995; Abo-Ollo et al., 2018; Hassan et al., 2022). Improving sugar beet resistance is essential to maintain crop production while reducing ecological side effects. Controlling RKNs in the infected sugar beet fields is therefore critical to growers. The available sugar beet-resistant varieties have a lesser yield and implement weaker matches to the susceptible varieties (Yu, 2001). Yet, their sowing diminishes the infestation intensity in plant parasitic nematode-contaminated soil by stimulating hidden J2 and inhibiting female development. Susceptible and resistant varieties are not tolerant to infection by infectious nematodes (Stevanato et al., 2015). According to as-yet-undiscovered processes, nematodes could work well regardless of whether they are infected or not. Although the acquisition costs of seeds are relatively high, growers tend to cultivate tolerant varieties since they reduce the risk of yield losses due to nematodes. Generally, the MHPI scale procedure detects tolerant sugar beet varieties more accurately because of the long test period that enables us to assess yield reduction.

The investigation of SNPs markers for sugar beet susceptible/resistance genotyping to RKNs was important. The use of allele-specific primers (ASPs), an SNPs marker with a single nucleotide polymorphism (A/G) that is associated with resistance genes, may help to differentiate resistant and susceptible genotypes (Zhao et al., 2021; Abbas et al., 2022; Alam et al., 2022). Also, the robust marker allowed consistent, faster, sensitive, and cheap large-scale assessment of *B. vulgaris* genotypes for breeding programs towards nematode resistance (Laurent et al., 2010; McGrath and Townsend, 2015; Ashmit et al., 2021).

In addition, Dwiningsih et al. (2020) described how the SNP marker had become the most common molecular marker for those studies. In addition, genes linked with the key yield characteristics could be identified more efficiently using the SNP marker compared to other molecular markers.

## Conclusion

The AQSCS and MHPI procedures can be used together when screening sugar beet for RKNs. The AQSCS was suitable for quick host suitability tests, especially for the susceptible varieties. The MHPI scale could be ranked as standardization of the host suitability method and reporting resistance or tolerance of sugar beet to RKNs. There are only a few publications on the genetic basis of nematode resistance of tolerant varieties. Here, we introduce high-yield genotypes that were tolerant or resistant to RKNs, which could help sugar beet breeders to produce new commercially desirable genotypes. Using the SNPs molecular markers was a crucial element in revealing the genetic variations among genotypes towards RKNs based on their genomic DNA sequences. This study provides promising results with prospects for understanding the molecular mechanisms of resistance against RKNs, which would help future challenges of developing effective practices that use eco-friendly, and sustainable disease management techniques.

## Data availability statement

The data that support the findings of this study are openly available in Figshare at https://doi.org/10.6084/m9.figshare.21664292.

## Author contributions

IG, NA, and AZ conceived and designed the research. IG, AZ, and NA supervised the study. KE-T, AA, MS, EM, RG, MH, and AZ assisted with data evaluation. IG, AA, MS, EM, RG, MH, KE-T and AZ analyzed the data. IG, NA, KE-T, and AE revised the manuscript. AA, MES and RYG contributed to statistical analysis, data visualization and validation; EMD contributed to grammar correction, English writing. All authors contributed to the article and approved the submitted version.
